# Characteristics of Carrier Transport and Crystallographic Orientation Distribution of Transparent Conductive Al-Doped ZnO Polycrystalline Films Deposited by Radio-Frequency, Direct-Current, and Radio-Frequency-Superimposed Direct-Current Magnetron Sputtering

**DOI:** 10.3390/ma10080916

**Published:** 2017-08-09

**Authors:** Junichi Nomoto, Katsuhiko Inaba, Shintaro Kobayashi, Takeshi Watanabe, Hisao Makino, Tetsuya Yamamoto

**Affiliations:** 1 Research Institute, Kochi University of Technology, Kochi 782-8502, Japan; makino.hisao@kochi-tech.ac.jp (H.M.); yamamoto.tetsuya@kochi-tech.ac.jp (T.Y.); 2 X-Ray Research Laboratory, Rigaku Corporation, Tokyo 196-8666, Japan; inaba@rigaku.co.jp (K.I.); s-kobaya@rigaku.co.jp (S.K.); 3 Japan Synchrotron Radiation Research Institute (JASRI), SPring-8, Hyogo 679-5198, Japan; t5511001@spring8.or.jp

**Keywords:** carrier transport, crystallographic orientation, initial growth stage, transparent conducting oxide, X-ray diffraction, Al-doped ZnO, magnetron sputtering

## Abstract

We investigated the characteristics of carrier transport and crystallographic orientation distribution in 500-nm-thick Al-doped ZnO (AZO) polycrystalline films to achieve high-Hall-mobility AZO films. The AZO films were deposited on glass substrates at 200 °C by direct-current, radio-frequency, or radio-frequency-superimposed direct-current magnetron sputtering at various power ratios. We used sintered AZO targets with an Al_2_O_3_ content of 2.0 wt. %. The analysis of the data obtained by X-ray diffraction, Hall-effect, and optical measurements of AZO films at various power ratios showed that the complex orientation texture depending on the growth process enhanced the contribution of grain boundary scattering to carrier transport and of carrier sinks on net carrier concentration, resulting in the reduction in the Hall mobility of polycrystalline AZO films.

## 1. Introduction

Polycrystalline materials consist of grains of finite size. The boundary between two grains is a lattice defect, across which the orientation of a crystal changes. Most polycrystalline Al-doped ZnO (AZO) films with a columnar grain structure show no strong out-of-plane texture and random in-plane orientation. An important parameter is, thus, the statistical distribution of the orientation of the out-of-plane texture. It can be affected by the film-growth methods mentioned below, their processes and/or deposition steps such as a multideposition process using a buffer layer and postannealing. The advantage of a polycrystalline AZO film over a single crystal AZO film, which has one continuous crystal because its structure contains no grain boundaries, is that the AZO film for large-size applications can be easily produced using several types of deposition methods such as magnetron sputtering [1,2,3,4,5,6], chemical vapor deposition [7], pulsed laser deposition [8,9,10], and the sol-gel method [11,12,13]. The polycrystalline structure has a completely different nature from that of a single crystal one. Columnar grains are not well aligned: most of the grains have the *c*-axis orientation aligned within a fraction of a degree of the film normal. The textured polycrystalline films can have different orientations such as (0001) and $\left(10\bar{1}1\right)$ orientations among the columnar grains. The disordered nature of the grain boundaries and the discontinuities that they introduce in the periodic structure of grains can directly reduce the electric current flow. In addition, we must take into account the effect of the grain boundaries on doping using external dopant atoms, that is, the change in the number of active *n*-type dopant atoms, Al donors, and free carriers in crystallites by acting as sites for dopant segregation and carrier trapping [14]. For such polycrystalline films, an approach to investigating the factors limiting carrier transport from a viewpoint different from those of conventional studies of single crystals is required. The study of the relationship between the carrier transport and the distribution of crystallographic orientations of transparent conductive polycrystalline AZO films [14,15,16,17,18] is, thus, of great importance from both viewpoints of fundamental materials science on the structural factors limiting carrier transport and practical technology how to achieve the carrier mobility required by the applications including electrical and optoelectronic devices.

Carrier transport is limited by two factors: the intrinsic carrier mobility in intragrains and the contribution of grain boundary scattering to the carrier transport governed by the crystallographic orientation distribution [14,15,16,17,18]. The degree of preferential orientation in a polycrystalline film with a columnar structure with a dominant orientation may vary widely from low for a weak fiber texture to high for a strong one. The texture of a fiber indicates the statistical distribution of grain orientations and is characterized by the alignment of planes in the film. For a fiber-textured polycrystalline film [14,15,16,17,18], one crystallographic axis of the film is parallel to the substrate normal, while there is a rotational degree of freedom around the fiber axis. In previous studies [14,15,16,17,18], we reported the difference in orientation distributions between direct-current (DC), radio-frequency (RF), and RF-superimposed DC (RF/DC) magnetron-sputtered AZO films. The RF-magnetron-sputtering technique produced 500-nm-thick AZO films having a strongly fiber-textured polycrystalline structure, which had most of the grains with the *c*-axis orientation aligned within a fraction of a degree of the film normal. On the other hand, we found that 500-nm-thick AZO films deposited by DC magnetron sputtering showed poor *c*-axis alignment between columnar grains, having textures with mixed orientations of the atomically closely packed (0001) and $\left(10\bar{1}1\right)$ planes. The formation of a columnar structure in a thin film with a thickness within a few tens of nanometers should be bound to the tendency of a system to diminish its surface free energy between the main crystallographic lattice planes of ZnO with the hexagonal structure: the magnitudes of surface free energy for the (0001), $\left(10\bar{1}1\right)$, and $\left(10\bar{1}0\right)$ planes are −2.8102, −2.1067, and −2.0013 kJ/mol, respectively [19,20]. 

The texture should determine structural, electrical, piezoelectric, and optical properties. The tailoring of orientation is critical for obtaining properties that meet the requirements for certain applications. Birkholz et al. showed that the sharpness of orientation distribution reduces the electrical resistivity of AZO films deposited by reactive DC magnetron sputtering from a metallic Zn-Al (2 wt. %) alloy target [21]. Gardeniers et al. demonstrated the relationship between the piezoelectric strain constant and the *c*-axis orientation of ZnO films prepared by reactive RF magnetron sputtering [22]. In our previous work [15], for 500-nm-thick AZO films grown by various types of magnetron sputtering with an AZO target (content of Al_2_O_3_, 2.0 wt. %), we obtained the following findings: AZO films deposited by DC magnetron sputtering had high optical mobility (*μ*_opt_), corresponding to high in-grain carrier mobility, high carrier concentration, and a large contribution of grain boundary scattering to carrier transport compared with AZO films deposited by RF magnetron sputtering; the Hall mobility (*μ*_H_) was reduced owing to the above-mentioned large contribution of grain boundary scattering to carrier transport for DC-magnetron-sputtered AZO films, whereas AZO films deposited by RF magnetron sputtering exhibited *μ*_H_ close to *μ*_opt_ owing to the little contribution of grain boundary scattering to carrier transport. Here, the contribution of grain boundary scattering to carrier transport is defined as a ratio of *μ*_opt_ to the carrier mobility at grain boundaries (*μ*_GB_), *μ*_opt_/*μ*_GB_, on the basis of Matthiessen’s rule [14,15,16,17,18].

In this study, we attempted to more accurately determine the crystallographic orientation distribution in AZO films deposited by DC magnetron sputtering or RF magnetron sputtering together with RF/DC magnetron sputtering at various power ratios, to investigate the relationship between the crystallographic orientation distribution and carrier transport for the determination of factors except for carrier concentration limiting the carrier transport of polycrystalline AZO films [23,24].

## 2. Results and Discussion

### 2.1. Hall Mobility and Carrier Concentration

The investigation of the relationship between *μ*_H_ and carrier concentration (*N*) is, thus, very important for clarifying the factors limiting carrier transport [23,24]. Polycrystalline films consist of grains and grain boundaries corresponding to the interface between two grains. Such defects, due to the disordered nature of the grain boundaries of the films showing a texture with poor alignment between columnar grains and the discontinuities that they introduce into the periodic structure of the grains of the films having a texture with a mix crystallographic orientation, can act as barriers for transport, resulting the creation of a potential barrier for free electrons, and can have a large impact on carrier transport together with *N*. This high and narrow grain-boundary barrier is assumed to be present in addition to the parabolic, depletion-region barriers surrounding the grain boundary. Hence, even though a very high carrier concentration leads to a reduction in the height and width of the above potential barrier, *μ*_H_ for the film where the additional potential barrier due to the structural defects is not well below the Fermi level may differ significantly from the carrier mobility affected by several carrier-scattering mechanisms in grains [23,24,25,26,27,28,29]. According to Seto’s theory [25], the grain boundary scattering to carrier transport is dominant for polycrystalline films with *N* in the range from 10^19^ to 10^20^ cm^−3^. With further increasing *N*, assuming that all the grain-boundary states are filled with trapped carriers and the electron charge trapped at the grain boundary cannot increase any more, the space charge density in the depletion region increases, leading to a reduction in the height and width of the parabolic, depletion-region potential barriers created by the uncompensated dopant atoms that neutralize carriers trapped at the grain boundary and making it transparent to carrier tunneling thorough the potential barrier. As a result, ionized impurity scattering mechanism in grains [30,31,32,33,34] mainly limits the carrier transport for a highly doping level; *μ*_H_ is close to intrinsic mobility in the grains. In the basic carrier-trapping model, the grain boundaries in polycrystalline films are assumed to be very narrow compared to the grains. Their effect is to change the number of the electrically active dopant atoms and resulting free carriers in the crystallites by acting as sites for dopant segregation and carrier trapping. For the conventional trapping model, the parabolic, depletion-region potential barriers would be created by the uncompensated dopant atoms that neutralize carriers trapped at the grain boundary. Talking into the account that real polycrystalline films have disordered nature of the grain boundaries and the discontinuities that they introduce into the periodic structure of the grains, a high and narrow energy barrier at the grain boundary would be formatted, in addition to the energy barrier described above. For the case of the composite energy barrier being wide and high, we consider thermionic-field emission, in which carriers are thermally excited before tunneling thorough a portion of the energy barrier. The specific energetic distribution of the traps at grain boundaries might explain the *μ*_H_ drop at high *N* by thermionic field emission mechanism [35]. On the other hand, for very high *N*, at which the parabolic, depletion region energy barriers are small, the added energy barriers at the grain boundaries would mainly limit the carrier transport. In such films with the carriers having sufficient energy, i.e., high Fermi energy, to surmount the parabolic energy barrier in the depletion regions but not sufficient energy to travel over the grain-boundary energy barrier: the carrier transport occurs by tunneling thorough the grain-boundary energy barrier. The above films will correspond to AZO films with Proc.s 2, 3 and 4 as shown in Table 1. Note that the conventional carrier transport studies mentioned above [23,24,25,26,27,28,29] lack information on crystallographic orientation distribution. The findings of those studies [23,24,25,26,27,28,29] are reasonable for ZnO-based conductive films having a fiber texture with a well-defined (0001) orientation regardless of *N* and deposition methods. Note that there is good reason to assume that the grain boundaries are characterized by a surface density *N_t_* of grain-boundary traps with an energy *E_t_* and all these traps occur at the same energy *E_t_*. In real polycrystalline films with a columnar grain structure, the two crystal grains that have the same or different orientations meet each other with a relative tilt and/or twist. We found that AZO films deposited by DC magnetron sputtering have (0001) mixed with $\left(10\bar{1}1\right)$ orientations. *N_t_* of the interface between two crystallites with the same orientation would be different from that of the boundary between two crystallites with the different orientation each other. Note that there is no good reason for the assumption described above for the films. In such films, we would find some distributions of trap energies throughout the band gap. This implies that the contribution of grain boundary scattering to carrier transport should depend on the degree of alignment between columnar grains and the crystallographic orientation distribution together with *N*.

**Figure 1:**
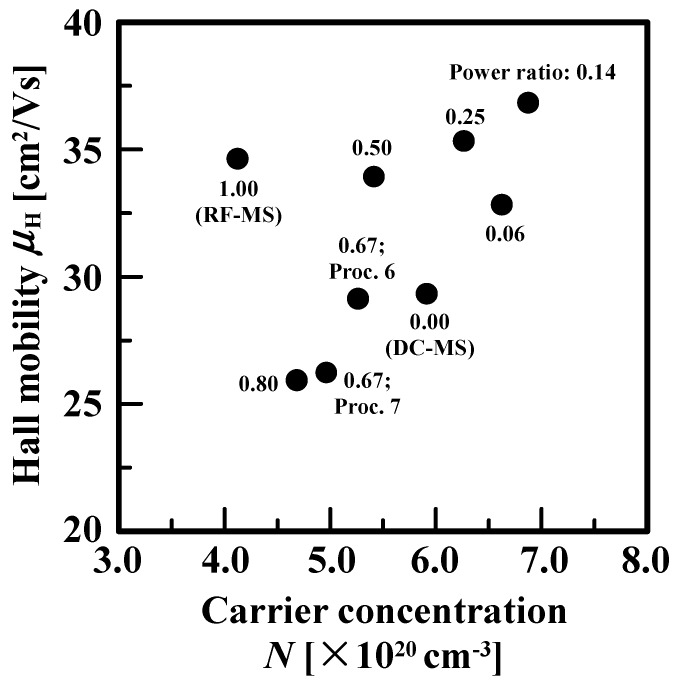
Hall mobility (*μ*_H_) vs. carrier concentration (*N*) of 500-nm-thick AZO films deposited by DC magnetron sputtering (DC–MS), RF/DC magnetron sputtering at various power ratios, and by RF magnetron sputtering (RF–MS).

Figure 1 shows *μ*_H_ as a function of* N* of 500-nm-thick AZO films deposited by DC magnetron sputtering, RF/DC magnetron sputtering at various power ratios, and RF magnetron sputtering. The nine different deposition processes carried out at various power ratios are summarized in Table 1 [15]. DC magnetron sputtering (Proc. 1) and RF magnetron sputtering (Proc. 9) were conducted at a DC power (P_DC_) of 200 W and an RF power (P_RF_) of 200 W, respectively. The RF/DC magnetron sputtering processes, from Proc. 2 to Proc. 8, were carried out by adding an RF component in the power range from 10 to 200 W to an applied P_DC_ in the power range from 50 to 150 W. Details of the various power ratios of P_RF_ to the total power (P_RF_ + P_DC_) are given in Table 1. From Figure 1, we found that an increase in *N* appear to tend to an increase in *μ*_H_ of AZO films except for AZO films deposited by RF magnetron sputtering. Note that AZO films deposited at a power ratio of 0.14 showed the highest *N* and *μ*_H_. Note that the averaged Al concentrations in depth for each of the AZO films were estimated from about 6.2 × 10^20^ to 6.6 × 10^20^ atoms/cm^−3^ on the basis of analysis of the data determined by secondary ion mass spectrometry (SIMS) measurements. The concentration of Al donors in the AZO films was found to remain almost constant from the film/substrate interface to the surface, regardless of the deposition process. Those findings implies that the above change in *N* would be due to the dependency of carrier activation efficiency of Al donors in grains and/or incorporation of Al atoms in crystallites and at grain boundaries, which would affect the carrier transport in polycrystalline films, on the RF/DC power ratios. 

**Figure 2:**
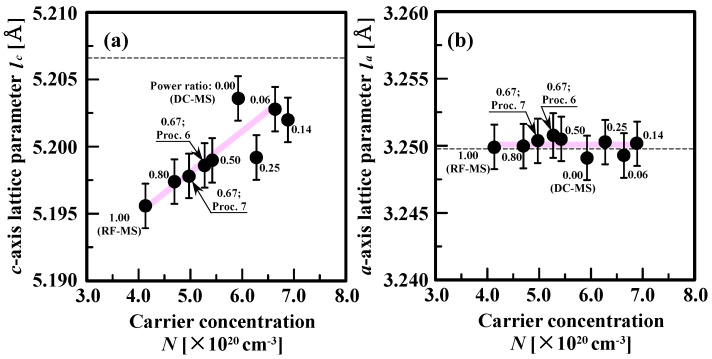
Relationship between (**a**) *c*-axis lattice parameter (*l*_c_) or (**b**) *a*-axis lattice parameter (*l*_a_) and carrier concentration (*N*) of 500-nm-thick AZO films deposited by DC magnetron sputtering (DC–MS), RF/DC magnetron sputtering at various power ratios and by RF magnetron sputtering (RF–MS).

Next, we investigated the dependence of lattice parameters, namely *c*-axis lattice parameter (*l_c_*) and *a*-axis lattice parameter (*l_a_*), on *N* of AZO films deposited at different power ratios, as shown in Figure 2. *l_c_* and *l_a_* values of AZO films were calculated from the 0002 reflection peak positions in the out-of-plane *θ*/2*θ* XRD profile and from the $10\bar{1}0$ reflection peak positions in the in-plane XRD profile [15]. The broken lines in Figure 2 indicate the values of the *l_c_* and *l_a_* for undoped ZnO powder. In this study, we assume that the Al doping provides electron states directly well inside the conduction band of ZnO films, which is mainly characterized as the Zn *4s* states with the Zn-O antibonding character. This means that an Al ion acts as an electron donor and its ionic valence becomes Al^3+^. The incorporation of Al donors has two distinct effects on the lattice parameters. The first effect is the size effect, which is related to the difference in ionic radius between Al species and the host Zn atom, which is replaced by Al dopants. The ionic radii of Al^3+^ and Zn^2+^ with a coordination number, i.e., the number of nearest-neighbor ions of opposite charge, of 4 are 0.39 and 0.60 Å, respectively. The second effect is the electronic effect, which is related to deformation potentials. Figure 2 shows that *l_c_* monotonically increases from 5.1956 to 5.2036 Å with increasing *N*, whereas *l_a_* changes negligibly; this behavior may be due to the strong adhesion of AZO films with the substrates. The occupation in the antibonding state likely results in repulsive forces between Zn and O, which lower the total energy of the crystal structure, thereby inducing expansion of the lattice. Analysis of data obtained by ab initio electronic band structure calculations based on the density functional theory within the generalized gradient approximation using VASP software [36] shows that the average bond length of Al-O over the four bond directions of AZO crystals with an Al content of 1 at.% is 1.80 Å. This is close to that (1.78 Å) of the sum of the ionic radii of Al^3+^ and O^2−^ with a coordination number of 4 (1.38 Å). On the other hand, the average bond length along the *c*-axis between O and Zn locating at the second-nearest neighbor of Al replacing Zn atoms, the total number of which is 12, is 2.04 Å, which is larger than 1.98 Å of the Zn-O bond length in undoped ZnO crystal, resulting in a larger *l_c_* than that of undoped ZnO crystal. Both theoretical and experimental results indicated that the *n*-type dominant defect of AZO films was Al substituting Zn atoms. Note that with increasing *N*, *l_c_* increases (see Figure 2a) and *l_a_* changes little (see Figure 2b), resulting that the unit cell volume *V* (∝ *l_a_* × *l_a_* × *l_c_*) increases. This can lead to a shift in the energy position of the conduction-band minimum towards the low energy region, with a proportionality factor given by the deformation potential, resulting in the reduction in the energy of the system. Taking into account the finding that the acoustic deformation potential scattering is not dominant in polar ZnO semiconductors [29], the change in volume following the incorporation of Al donors should have a negligible effect on *μ*_H_, as shown in Figure 1. Therefore, we focused on the effects of crystallographic orientation distribution on the carrier transport of magnetron-sputtered AZO films deposited at different power ratios.

These discussion of the Al addition on the lattice constant of their films are based on the ionic radii. Ellmer et.al reported that the covalent radii of the dopants describe the doping effects of group III dopants better than the ionic radii [37]. Furthermore, the dependence of the lattice constant on the *N* also was discussed in detail, taking into account the effect of the added dopant amount: solubility limit of Al in ZnO, according to Vegard rule [38] and a possible role of intrinsic *n*-type and *p*-type defects. More quantitative discussions from both sides of theory and experimental are required.

### 2.2. Texture Evolution

**Figure 3:**
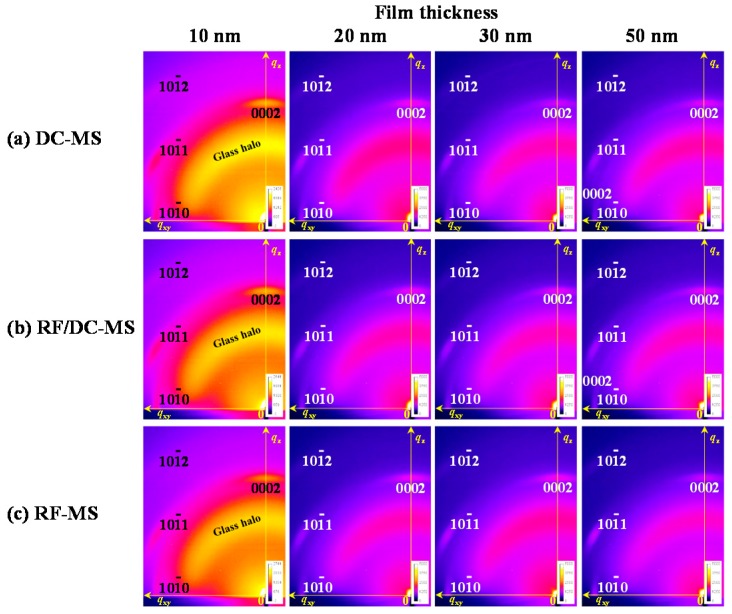
Two-dimensional diffraction images of Al-doped ZnO (AZO) films with film thicknesses of 10, 20, 30, and 50 nm deposited by (**a**) DC magnetron sputtering (DC–MS), (**b**) RF/DC magnetron sputtering (RF/DC–MS) at a power ratio of 0.14, and by (**c**) RF magnetron sputtering (RF–MS).

**Figure A2:**
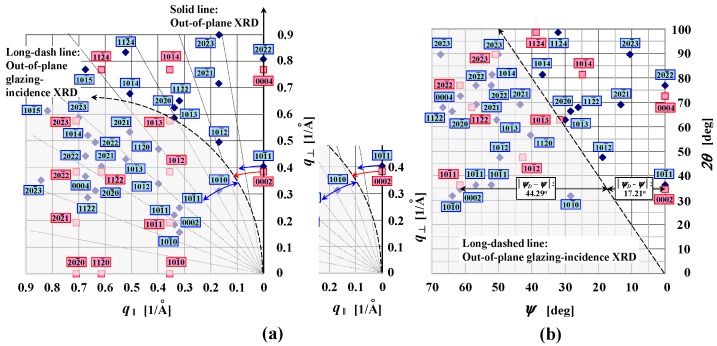
Reciprocal space map simulation results drawn with (**a**) *q* coordinate system and (**b**) goniometer coordinate system for ZnO films having a texture with mixed orientations of atomically closely packed (0001) and $\left(10\bar{1}1\right)$ planes.

We characterize the evolution of the crystallographic orientation of AZO films with various thicknesses ranging from 10 to 50 nm deposited by DC, RF/DC, or RF magnetron sputtering at various power ratios. Figure 3a–c show the two-dimensional diffraction images of AZO films with thicknesses of 10, 20, 30, and 50 nm deposited by DC magnetron sputtering and RF/DC magnetron sputtering at a power ratio of 0.14, and by RF magnetron sputtering, respectively. *q*_z_ and *q*_xy_ are the stacking direction and in-plane direction, respectively. Signals of multiple reflections on the *q*_z_ axis in Figure 3 are these from crystallites being tilted and deviated from the surface normal direction. More details are given in a previous technical article [39]. All figures show peaks of the $10\bar{1}2$, $10\bar{1}1$, and $10\bar{1}0$ reflections and 0002 reflection on the *q*_z_ axis, which originated from the (0001) orientation, even though the 10-nm-thick AZO films were very thin. These results clearly showed that AZO films had a preferential (0001) orientation texture at the very early stage of their growth. We, however, assumed that both the DC- and RF-superimposed-DC magnetron-sputtered AZO films would include some crystallites with a very small amount of $\left(10\bar{1}1\right)$ orientation in addition to the (0001) orientation at the early stage of their growth. This assumption is based on the following experimental results on the evolution of several reflections observed for AZO deposited by DC magnetron sputtering and AZO films deposited by RF/DC magnetron sputtering as follows. We found little difference in two-dimensional diffraction images among AZO films deposited by RF magnetron sputtering, DC magnetron sputtering, and RF/DC magnetron sputtering at any given thickness ranging from 10 to 30 nm. On the other hand, in case of 50 nm thicknesses, we found distinct differences in two-dimensional diffraction images among the RF-, DC- and RF-superimposed-DC magnetron-sputtered AZO films: Figure 3a,b presented that AZO films deposited by DC magnetron sputtering or RF/DC magnetron sputtering showed another 0002 reflection. The 0002 reflection corresponds reasonably well with the reciprocal space map simulation in Figure A2a. This means that the two different types of 50-nm-thick AZO films consistently showed a unique texture with the $\left(10\bar{1}1\right)$ orientation in addition to the (0001) orientation. On the other hand, for the RF-magnetron-sputtered AZO films shown in Figure 3c, we found no significant difference in the reflection images among the films with thicknesses ranging from 10 to 50 nm. Therefore, we concluded that 10-nm-thick AZO films deposited by RF magnetron sputtering showed a highly textured (0001) orientation. The characteristics of the above orientation distribution of RF-magnetron-sputtered AZO films with increasing thickness up to 50 nm should be retained with further increasing thickness.

**Figure 4:**
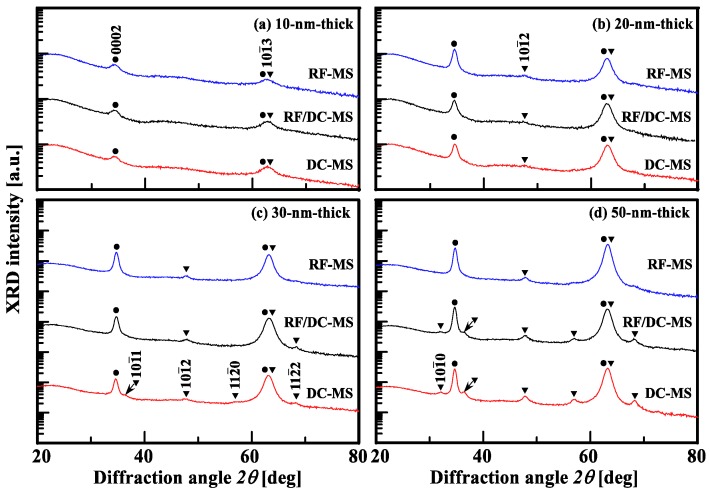
Out-of-plane grazing-incidence XRD patterns of (**a**) 10, (**b**) 20, (**c**) 30, and (**d**) 50-nm-thick AZO films deposited by DC magnetron sputtering (DC–MS) and RF/DC magnetron sputtering (RF/DC–MS) at a power ratio of 0.14, and RF magnetron sputtering (RF–MS).

We carried out out-of-plane grazing-incident XRD measurements [15,16,17,18] to demonstrate a significant difference in the film growth among AZO films deposited by the three different types of magnetron sputtering methods mentioned above. Figure 4 shows the out-of-plane grazing-incident XRD patterns of the same films as those shown in Figure 3. The solid black circles (●) and the solid black inverted triangles (▼) correspond to the contributions of (0001) and $\left(10\bar{1}1\right)$ orientations, respectively. The origins of reflections observed in the out-of-plane grazing-incident XRD measurement results (Figure 4) can be determined as follows: (I) the 0002 reflection, which corresponds to a component that originated from the (0001) orientation, can be observed owing to the tilting of the *c*-axis, as shown on the right side of Figure A2a; (II) $10\bar{1}0$, $10\bar{1}1$, $10\bar{1}2$, $11\bar{2}0$, and $11\bar{2}2$ reflections are components that originated from the $\left(10\bar{1}1\right)$ orientation owing to the tilting of columnar grains with the $\left(10\bar{1}1\right)$ orientation; (III) the $10\bar{1}3$ reflection corresponds to the trajectory of ***Q***, as shown in Figure A2a, when films have a (0001) orientation and/or an $\left(10\bar{1}1\right)$ orientation.

As shown in Figure 4a, the analysis of the data obtained by the out-of-plane grazing-incident XRD measurements of 10-nm-thick AZO films deposited by DC magnetron sputtering, RF/DC magnetron sputtering or RF magnetron sputtering showed predominant 0002 and $10\bar{1}3$ reflections. Figure 4b showed that with increasing thickness up to 20 nm, the AZO films exhibited also a barely resolvable $10\bar{1}2$ reflection together with the 0002 and $10\bar{1}3$ reflections regardless of the deposition process. From Figure 4c,d, we found that with increasing thickness up to 50 nm, RF-magnetron-sputtered AZO films retained the above-described three reflections: the AZO films with different thicknesses had a common feature of well-defined (0001) orientation. On the other hand, for 50-nm-thick AZO films deposited by DC magnetron sputtering or RF/DC magnetron sputtering, the $10\bar{1}0$, $10\bar{1}1$, $11\bar{2}0$, and $11\bar{2}2$ reflections were also observed in addition to the above-described three peaks. The two different types of AZO film may include a small number of crystallites with the $\left(10\bar{1}1\right)$ orientation in addition to those with the (0001) orientation at the early stage of film growth.

**Figure 5:**
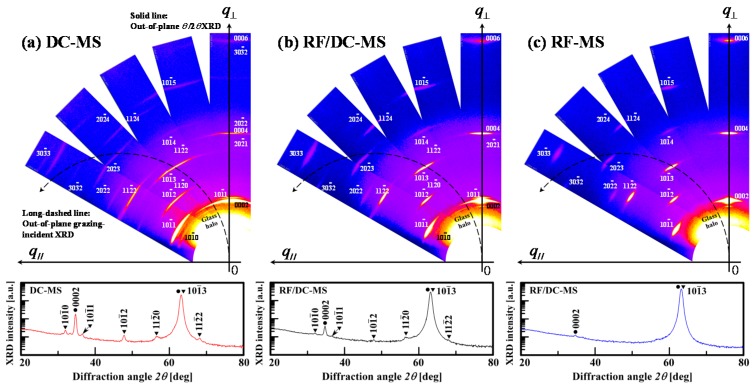
Wide-range XRD reciprocal space maps of 500-nm-thick AZO films deposited by (**a**) DC magnetron sputtering (DC–MS), (**b**) RF/DC magnetron sputtering (RF/DC–MS) at a power ratio of 0.14, and by (**c**) RF magnetron sputtering (RF–MS).

In the following, we investigate the characteristics of the orientation distribution of thick AZO films deposited by the different types of magnetron sputtering under consideration. Figure 5a–c show the measurement results obtained by wide-range XRD reciprocal space map measurements of 500-nm-thick AZO films deposited by DC magnetron sputtering and RF/DC magnetron sputtering at a power ratio of 0.14, and by RF magnetron sputtering, respectively. From Figure 5, we found that all AZO films have {0001} families of planes parallel to the substrate surface. *q*_//_ and $q_{⊥}$ are the coordinates of the reciprocal space (*q =* 1/*d_hkil_* = 2sin*θ*/*λ*, *θ* and *λ* are the incident angle and the wavelength of X-rays, respectively); *q*_//_ is in the direction parallel to the surface and $q_{⊥}$ is in the direction perpendicular to the surface. The solid line and the long-dashed line in the reciprocal space maps correspond to an orbital of a symmetric *θ/*2*θ* coupled scan of out-of-plane *θ*/2*θ* XRD measurements (see Figure 2a,c,i of Ref. [15]) and to that of a *ω*-fixed 2*θ* scan of out-of-plane grazing-incident XRD measurements, which are shown in the insets of Figure 5. For AZO films deposited by DC magnetron sputtering shown in Figure 5a, the analysis of the data obtained from XRD reciprocal space maps revealed peaks of the $10\bar{1}1$, $20\bar{2}1$, and $30\bar{3}2$ reflections together with those of the 0002, 0004, and 0006 reflections on a symmetrical zone in XRD reciprocal space maps. Note that the center of gravity of the peak of the $10\bar{1}1$, $20\bar{2}1$, and $30\bar{3}2$ reflections is observed to locate on the on $q_{⊥}$ axis in the XRD reciprocal space maps. This proves that the DC-magnetron-sputtered AZO films had a texture with the $\left(10\bar{1}1\right)$, $\left(20\bar{2}1\right)$ and $\left(30\bar{3}2\right)$ orientations. For AZO films deposited by RF/DC magnetron sputtering, from Figure 5b, we found not only the arc of each reflection narrowing but also multiple reflections diminishing. On the other hand, Figure 5c shows that AZO films deposited by RF magnetron sputtering consisted of columnar grains with a nearly perfect *c*-axis orientation close to a fiber *c*-axis orientation. Note that Figure 4d and Figure 5 shows an insignificant difference between the out-of-plane grazing-incident XRD patterns of 50- and 500-nm-thick AZO films: the growth stage of 50-nm-thick AZO films determines the final structure of the 500-nm-thick films deposited by DC magnetron sputtering or RF/DC magnetron sputtering, whereas that of 10-nm-thick AZO films deposited by RF magnetron sputtering should govern the resulting structure of 500-nm-thick films.

### 2.3. Correlation between Texture Evolution and Carrier Transport

**Figure 6:**
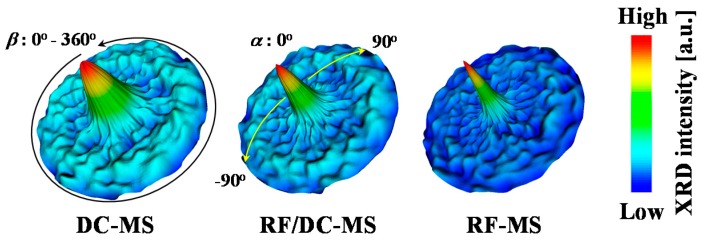
Three-dimensional projections of XRD pole figures of 0002 reflection of 500-nm-thick AZO films deposited by DC magnetron sputtering (DC–MS), RF/DC magnetron sputtering at a power ratio of 0.14 and by RF magnetron sputtering (RF–MS).

Figure 6a–c shows three-dimensional projections of XRD pole figures of the 0002 reflection in the same films as those shown in Figure 5, i.e., 500-nm-thick AZO films deposited by DC magnetron sputtering and RF/DC magnetron sputtering at a power ratio of 0.14, and by RF magnetron sputtering, respectively. For the analysis of the texture of AZO films with columnar grain structures, we focused on the *c*-face (0001) distribution of the AZO film, since the *c*-axis of AZO is unique in the wurtzite structure. In Figure 6, the distribution of the poles for 0002 reflections was observed as a spot at the center of the figure or as two rings with uniform intensity. This demonstrated that the textures of the AZO films are isotropic in terms of rotation around the surface normal direction. In general, the angle magnitude (*ψ*_(*hkil*)_) between the normal of (0001), i.e., the *c*-axis and the normal of any diffraction planes (*hkil*) provides us with a clear understanding of the origin of the peaks at various intensities. Figure 6 clearly shows two peaks located at *α* values of 0° and 66° (denoted hereafter by the first and second peaks, respectively). The first peak was attributed to the (0001) orientation [14,17,18]. The presence of the second peak revealed that AZO films have a mixture of multiple orientations, such as $\left(10\bar{1}1\right)$ [40], $\left(30\bar{3}2\right)$, and $\left(20\bar{2}1\right)$, for which *ψ*_(10–11)_, *ψ*_(30–32)_, and *ψ*_(20–21)_ are 61.58°, 70.16° and 74.86°, respectively [14,17,18]. 

**Figure 7:**
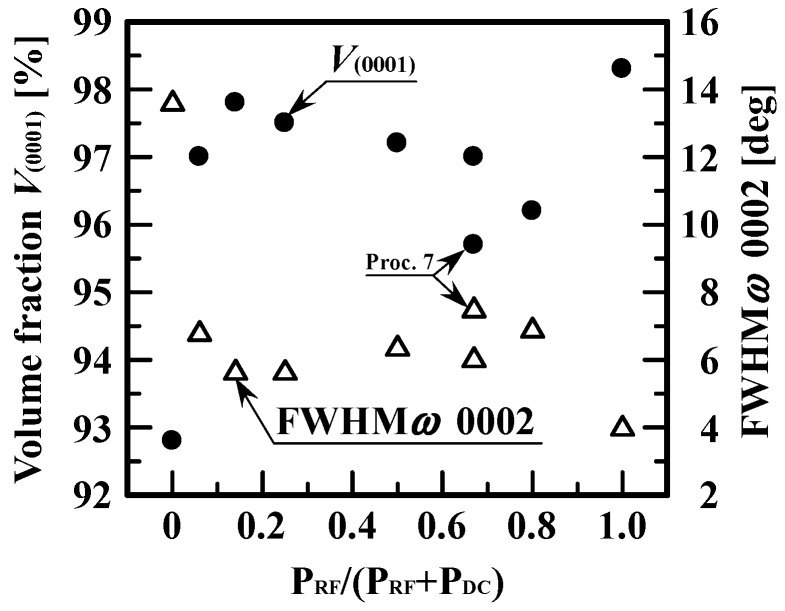
Volume fraction of grains with the (0001) orientation (*V*_(0001)_) and the values of the full width at half maximum (FWHM) of the *ω* rocking curves of the 0002 reflection of AZO films as a function power ratio, P_RF_/(P_RF_ + P_DC_).

To characterize the degree of the (0001) orientation, we estimated the volume fraction of grains with the (0001) orientation, *V*_(0001)_; the larger the value of *V*_(0001)_, the higher the degree of the (0001) orientation. Figure 7 summaries *V*_(0001)_ of 500-nm-thick AZO films deposited at various power ratios. Figure 7 also summaries the values of the full width at half maximum (FWHM) of the *ω* rocking curves of the 0002 reflection of the same films above, as a reference [15]. In Figure 7, for the AZO films deposited at power ratios from 0.06 to 0.8, the values of *V*_(0001)_ were much higher than those of AZO films deposited at a power ratio of 0.00, corresponding to AZO films deposited by DC magnetron sputtering. The above analysis showed that the RF/DC magnetron sputtering technique is an effective way of improving the (0001) orientation of AZO films with columnar grains. Note that at a power ratio of 1.0, *V*_(0001)_ increased abruptly: the AZO films deposited by RF magnetron sputtering had a texture with a preferential (0001) orientation at the expense of the orientations of the others.

On the basis of the above findings, let us consider the relationship between *V*_(0001)_ and the carrier transport of AZO films deposited at various power ratios. In our previous work, we reported that the *μ*_opt_ of these films changed slightly with power ratio, whereas *μ*_H_ was mainly governed by the contribution of grain boundary scattering to carrier transport defined as the ratio of *μ*_opt_ to *μ*_GB_ (*μ*_opt_/*μ*_GB_); *μ*_opt_/*μ*_GB_ strongly depended on power ratio [14,15,16,17]. For the AZO films having a texture with a well-defined (0001) orientation, the degree of alignment between columnar grains would predominantly determine *μ*_opt_/*μ*_GB._ In such films, taking into account the finding that the peak of the 0002 reflection corresponds to a combination of planes to the surface, which consequently holds information about the out-of-plane misorientation of domains (tilt), the mean FWHM of the peak would be an important factor for the relationship between the orientation distribution and *μ*_opt_/*μ*_GB_. 

**Figure 8:**
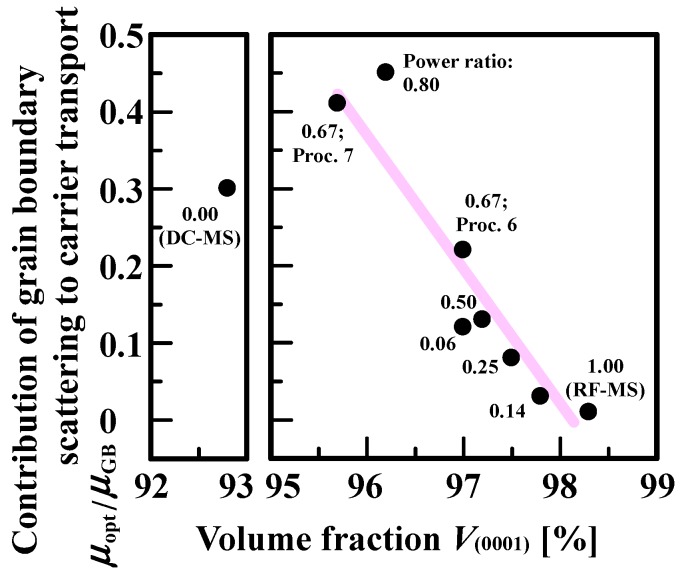
Contribution of grain boundary scattering to carrier transport (*μ*_opt_/*μ*_GB_) as a function of volume fraction of (0001) orientation (*V*_(0001)_) of 500-nm-thick AZO films deposited by DC magnetron sputtering (DC–MS) and RF/DC magnetron sputtering at various power ratios, and by RF magnetron sputtering (RF–MS).

In this study, we demonstrated the factor limiting the *μ*_opt_/*μ*_GB_ of AZO films having a texture with a mixed orientation deposited by different types of magnetron sputtering process. Figure 8 shows *μ*_opt_/*μ*_GB_ as a function of *V*_(0001)_ of the AZO films. From Figure 8, we found that *μ*_opt_/*μ*_GB_ exhibited a tendency to decrease with increasing *V*_(0001)_ except for the AZO films deposited by DC magnetron sputtering. This indicated that the presence of complex orientations such as $\left(10\bar{1}1\right)$, $\left(20\bar{2}1\right)$, and $\left(30\bar{3}2\right)$ should give rise to the increase in *μ*_opt_/*μ*_GB_, resulting in the reduction in *μ*_H_ of polycrystalline AZO films [14,15,16,17]. For processes 6, 7, and 8, which correspond to the power ratios of 0.67, 0.67, and 0.80, respectively, we found a large decrease in *V*_(0001)_, which should enhance the disordered nature of grain boundaries. The grain boundaries can be characterized by a high, narrow, potential barrier in addition to parabolic, depletion-region potential barriers created by the uncompensated dopant atoms that neutralize carriers trapped at the grain boundary. The above the disordered nature and discontinuities that they introduce in to the periodic structure of the crystallites would substantially decrease *μ*_GB_. Note that AZO films deposited by processes 6, 7, and 8 had the reduced *N* [15]: The grain boundaries possibly acted as carrier sinks. In contrast, AZO films with very low *V*_(0001)_ deposited by DC magnetron sputtering had higher *N* than the AZO deposited by the three different types of process, as shown in Figure 1 [15]. This should lead to the formation of a grain boundary potential barrier with a small energy difference relative to the Fermi level, resulting in a high *μ*_GB_. Taking into account the finding that the DC-magnetron-sputtered AZO films had *μ*_opt_ higher than the AZO films with the three different power ratios above [15], *μ*_opt_/*μ*_GB_ would be reduced, which was confirmed in Figure 8. These findings proved that *V*_(0001)_ is a dominant factor limiting the *μ*_opt_/*μ*_GB_ of AZO films having a texture with a mixed crystallographic orientation. 

Sato et al. showed that an undoped grain boundary is electrically inactive, whereas when some of dopant atoms are segregated at the grain boundaries, that leads to the formation of defect states and of energy barriers at grain boundaries in ZnO bicrystals [41]. Bikowski et al. reported that the grain boundary defects are not caused by crystallographic defects, but, most probably, by the dopant such as Al species [42]. Jia et al. explained that the trap density at the grain boundary increases with increasing amount of Al species in the films [43]. In this study, we demonstrated that increase of *V*_(0001)_ owing to the reduction in the others orientation except for (0001) orientation leads to an increase in the carrier activation of Al donors as a result of the improved whole crystallinity together with a decrease in the contribution of grain boundary scattering, which should be caused by the grain-boundary segregation of Al dopants, to carrier transport at grain boundaries.

## 3. Experimental Details

### 3.1. Film Deposition

We deposited 500-nm-thick AZO films on glass substrates (Corning Eagle XG, New York, NY, USA) at a substrate temperature of 200 °C by three different magnetron sputtering deposition methods: DC magnetron sputtering, RF magnetron sputtering and RF/DC magnetron sputtering. The oxide targets were high-density sintered circular AZO targets (diameter: 80 mm) prepared with an Al_2_O_3_ content of 2.0 wt. %. We used a magnetron-sputtering apparatus (ULVAC CS-L, Kanagawa, Japan) with a sintered oxide target. The nine different deposition processes using the various magnetron-sputtering techniques mentioned above are summarized in Table 1 [15]. DC magnetron sputtering corresponding to Proc. 1 and RF magnetron sputtering denoted by Proc. 9 were conducted with a P_DC_ of 200 W and a P_RF_ of 200 W, respectively. The RF/DC magnetron-sputtering processes, from Proc. 2 to Proc. 8, were carried out by adding an RF component in the power range of 10–200 W to an applied P_DC_ of 50–150 W. The deposition rate was changed power ratios, however, which was not a dominant factor determining the properties of the films in our experiment [15].

All deposition processes were performed out in a pure argon (Ar) atmosphere at a pressure of 1.0 Pa. Prior to the depositions, the chamber was evacuated until the base pressure reached about 2.0 × 10^−5^ Pa. The substrate was rotated at a velocity of 10 rotation per minute during the depositions. A substrate with an area of 100 × 100 mm^2^ was placed parallel to the target surfaces with a minimum substrate-target distance of 100 mm [15].

### 3.2. Characterization

The depth profiles of Al concentration in AZO thin films were determined by SIMS measurements. The textures of the films were characterized by measurements of wide-range out-of-plane reciprocal space maps [14,16,17,44] and pole figures [14,16,17,45,46,47,48] using the SmartLab XRD system (Rigaku Corp, Tokyo, Japan) equipped with a PILATUS 100 K/R two-dimensional X-ray detector using Cu-K$\bar{\alpha }$ radiation (wavelength *λ* = 0.15418 nm; weighted average of Cu-Kα_1_ *λ* = 0.154059 nm/Cu-Kα_2_ *λ* = 0.15444 nm with an intensity ratio of 2:1). The width of the X-ray beam on the samples is 10 mm during XRD measurements. Each pole figure was measured at a fixed scattering angle and by serial *β* scanning (azimuthal rotation around normal to the surface of the sample by 0° to 360°) at different tilts with *α* steps of 0 to 90°, which correlated with the angle of the scattering vector from the surface normal vector. Reciprocal space map measurements should have access to the lattice plane inclined at an angle of *ψ*. In general, owing to the geometrical restriction required to maintain the skew geometry (*θ*/2*θ* geometry), the sample was tilted about the *χ*-axis, while the two-dimensional detector was scanned in the time-delayed integration mode [44,49]. 

For a comprehensive analysis of the texture, we carried out two different methods of grazing-incident XRD measurement. One method is as follows: we carried out out-of-plane grazing-incident XRD measurements [14,16,17,18,49,50,51,52,53] with the ATX-G XRD system (Rigaku Corp, Tokyo, Japan) using Cu-K$\bar{\alpha }$ (wavelength *λ* = 0.15418 nm) radiation, where X-ray was irradiated on the substrate surface at an incident angle (*ω*) of 0.35° and only the 2*θ* axis was scanned. The other method is as follows: we took the two-dimensional diffraction images [54], that were obtained using a multiaxes diffractometer (HUBER Diffraktionstechnik GmbH & Co., KG, Rimsting, Germany) combined with a two-dimensional detector (PILATUS 300 K) at the BL19B2 beam line in SPring-8. The measurement technique is called grazing incident wide-angle X-ray scattering (GI-WAXS). The X-ray energy for this experiment was 12.40 keV (wavelength *λ* = 0.1 nm). The highly brilliant and collimated synchrotron X-ray was irradiated at *ω* = 0.15°. The diffracted X-rays were detected over an exposure time of 30 s. The distance between the sample and the detector was 174 mm, which was calibrated using a polycrystalline CeO_2_ [54]. The reason why we chose grazing-incident XRD analysis, particularly, two-dimensional diffraction imaging using a combination of a two-dimensional detector and a high-brightness synchrotron X-ray source, is to make it possible to analyze crystal structures in detail and the orientation distribution of the thinner films [54,55,56,57,58].

The lattice parameters of the AZO films were calculated by XRD analysis using Cu-K$\bar{\alpha }$ (wavelength *λ* = 0.15418 nm) radiation (Rigaku Corp., ATX-G). The out-of-plane *θ*/*2θ* XRD pattern (obtained by synchronous scanning during which X-ray incident beam angle (*ω*) was fixed at half of the diffracted beam angle (2*θ*)) and the in-plane XRD pattern (obtained by synchronous scanning of *2θχ* and *φ* in the azimuth plane during which *ω* and 2*θ* are fixed at 0.35°).

## 4. Summary

In this study, we examined the characteristics of AZO films deposited by DC magnetron sputtering, RF magnetron sputtering or RF/DC magnetron sputtering. The data obtained by Hall-effect measurements showed no clear relationship between *μ*_H_ and *N*. We found a correlation between structural and electrical properties; an increase in *N* led to an increase in *l*_c_. The conventional DC-magnetron-sputtering technique produced AZO films with a poor alignment between the (0001) oriented columnar grains owing to the presence of grains with the other orientations, such as $\left(10\bar{1}1\right)$, $\left(20\bar{2}1\right)$, and $\left(30\bar{3}2\right)$ orientations. On the other hand, AZO films deposited by the conventional RF magnetron sputtering showed a texture with a well-defined (0001) orientation; the AZO films had columnar grains exhibiting a preferential *c*-axis orientation. The RF/DC magnetron-sputtering technique applied at various ratios from 0.06 to 0.8 enabled us to achieve AZO films having the texture with various distributions of crystallographic orientation together with *N*. Analysis of the data of the statistical distribution of the orientation of the out-of-plane texture obtained by XRD measurements yielded that *V*_(0001)_ strongly depended on the processes used at various power ratios and revealed a clear correlation between *V*_(0001)_ and *μ*_opt_/*μ*_GB_ of AZO films. This showed that the presence of the complex orientation texture played an important role in the contribution of grain boundary scattering to carrier transport. In this study, we proved that the design of the distribution of the orientation of the out-of-plane texture would be an effective way of achieving high-Hall-mobility polycrystalline AZO films.

## Figures and Tables

**Table 1:** Process number, DC power (P_DC_), RF power (P_RF_), total power (P_RF_ + P_DC_), and ratio of P_RF_ to total power (P_RF_/(P_RF_ + P_DC_)).

Process Number	P_DC_ (W)	P_RF_ (W)	Total Power, (P_RF_ + P_DC_) (W)	Power Ratio, P_RF_/(P_RF_ + P_DC_)
Proc. 1	200	0	200	0.00
Proc. 2	150	10	160	0.06
Proc. 3		25	175	0.14
Proc. 4		50	200	0.25
Proc. 5	100	100	200	0.50
Proc. 6		200	300	0.67
Proc. 7	50	100	150	0.67
Proc. 8		200	250	0.80
Proc. 9	0	200	200	1.00

**Figure A1:**
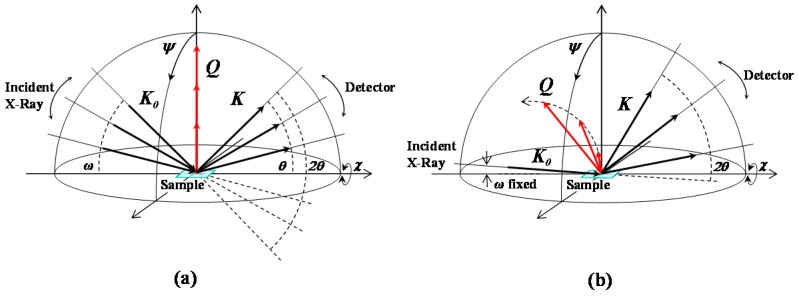
Schematic diagrams of out-of-plane (**a**) *θ*/2*θ* XRD and (**b**) grazing-incidence XRD geometries.

## Appendix A. Out-of-Plane Grazing-Incidence XRD Profiles Obtainable in AZO Films with (0001) and (10–11) Orientations

The working principle of out-of-plane *θ/*2*θ* XRD scanning is illustrated in Figure A1a. The wave vectors ***K*_0_** and ***K*** in Figure A1a describe the direction of the incoming and exiting X-rays, respectively. The sample is positioned at the center of the instrument and the probing X-ray is directed ward the sample surface at an angle ω. A symmetric *θ/*2*θ* coupled scan used to measure the Bragg diffraction angle is a plot of scattered X-ray intensity vs. 2*θ*. During the scanning, *ω* also changes in a 2*θ*-dependent manner that so that *ω* = ½ × 2*θ*. As a result of such out-of-plane *θ/*2*θ* XRD measurements, the scattering vector ***Q*** will be normal to the surface of the sample, as shown in Figure A2a. The symmetrical-reflection measurement is performed to collect diffracted X-rays from crystal lattice planes that are parallel to the sample surface throughout the entire measurement. This means that the out-of-plane *θ/*2*θ* XRD measurement is suitable for the analysis of the crystallographic information of the films with a strongly preferred orientation or an epitaxial film in the direction perpendicular to the sample surface.

Figure A1b schematically shows the configuration of the out-of-plane glazing-incidence XRD measurements. The angle between ***K*_0_** and the sample surface was very small (0.35°) in this study. Profiles are measured such that the angle *ω* is kept constant as the detector is moved along the 2*θ* circle. The detector scan (2*θ* scan) is an arc along the Ewald sphere circumference (Figure A1b), whereas the couple scan (the *θ/*2*θ* scan) is a straight line pointing away from the origin (Figure A1a). For out-of-plane grazing-incidence XRD measurements with a low angle of X-ray incidence, the 2*θ* scans with high sensitivity to textured polycrystalline thin films consisting of grains with a preferential orientation for three principal axes or only along one axis such as the *c*-axis can be used to probe several types of grains, which originate from mixed orientations with various tilt angles with respect to the substrate normal. Although out-of-plane grazing-incidence XRD measurements are powerful tools for collecting XRD signals from polycrystalline thin films, they are not practically convenient for the analysis of textured thin films, partly because the scattering vector ***Q*** is tilted from the surface normal vector and mainly because its direction is continuously changing during the 2*θ* scan. On the other hand, reciprocal space map simulations provide us with clear images of the distribution of diffraction spots of single-crystal-based substrates, epitaxial thin films, and also polycrystalline thin films. The out-of-plane grazing-incidence XRD analysis together with reciprocal space map simulations of textured polycrystalline thin films, therefore, can provide notable insights, such as the origin of the reflection peaks in relation to the orientation components, into thin films with complex orientation textures like our samples.

Figure A2a shows the reciprocal space map simulation results of ZnO with the (0001) orientation texture combined with the reciprocal space map simulation results of ZnO with the $\left(10\bar{1}1\right)$ orientation texture. The right figure, where only a representative area in the *q* coordinate system is focused on, in Figure A2a illustrates a broadening of the X-ray reflections for ZnO films with the tilt of the crystallites, giving rise to a broadening along circular paths (red and blue directional solid lines) around the origin. The gray hemisphere zones in Figure A2a indicate the areas where the configuration of a goniometer is 2*θ < ω* or *ω* < 0°. These configurations are the transmission geometry, and, are thus not suited for the XRD measurements of thin-film samples, in general. The limits of accessibility are, thus, the Laue zones (2*θ > ω* and *ω* > 0°). The red squares and blue diamonds correspond to representative reflections of the ZnO thin films with (0001) and $\left(10\bar{1}1\right)$ orientations, respectively. The sets of four numbers in the red and blue squares denote the *hkil* indices of reflections of the ZnO thin films with (0001) and $\left(10\bar{1}1\right)$ orientation textures, respectively. The solid and long-dashed directional lines correspond to orbitals for the out-of-plane *θ*/2*θ* XRD and glazing-incidence XRD scans, respectively. The orbital of ***Q*** passes multiple reflections caused by the mixed orientations. Figure A2a, thus, shows that the trajectory of ***Q*** passes the 0002, $10\bar{1}1$, 0004, and $20\bar{2}2$ reflections in the case of the out-of-plane *θ*/2*θ* XRD, whereas the orbital of ***Q*** passes only the $10\bar{1}3$ reflection in the case of the out-of-plane grazing-incidence XRD.

To better understand the obtainable reflection peaks for textured polycrystalline AZO films with the mixed orientations under consideration obtained by out-of-plane grazing-incidence XRD measurements, we convert the reciprocal space map simulation results from the *q* coordinate system (*q*_//_ and $q_{⊥}$) (Figure A2a) to the goniometer (*2θ* and *ψ*) coordinate system (Figure A2b). The relationship between the two coordinate systems is as follows: *q*_//_ = (1/*d*) × sin*ψ* and $q_{⊥}$ = (1/*d*) × cos*ψ,* where *d* is the interplanar spacing of the crystals or films, in accordance with Bragg’s law; 1/*d* = 2sin*θ*/*λ*. We, then, calculated the magnitudes of difference angles (|*ψ_D_ − ψ*|) between the diffraction peak (*ψ_D_*) caused by the (0001) and $\left(10\bar{1}1\right)$ orientations and the trajectory of out-of-plane grazing-incidence XRD (*ψ*). Table A1 summarizes the calculation results. Note that the definition of |*ψ_D_ − ψ*| provides that the smaller the magnitude, the stronger the trajectory with the diffraction peak corresponding to the orientation plane that would overlap with a small-tilted orientation plane. For example, for an obtainable 0002 reflection in the out-of-plane grazing-incidence XRD measurements, the |*ψ_D_ − ψ*| values caused by the (0001) and $\left(10\bar{1}1\right)$ orientations are 17.21° and 44.29°, respectively, as shown in Table A1. In this case, the intensity of the 0002 reflection that originated from the (0001) orientation is much higher than those caused by the $\left(10\bar{1}1\right)$ orientation. Table A1 indicates that the $10\bar{1}0$, $10\bar{1}1$, $10\bar{1}2$, $10\bar{1}4$, $10\bar{1}5$, $11\bar{2}0$, $11\bar{2}2$, $20\bar{2}0$, and $20\bar{2}1$ reflections are expected to be observed for the out-of-plane grazing-incidence XRD measurements of textured AZO films with the $\left(10\bar{1}1\right)$ orientation. Those reflections are shown in Figure 4 and Figure 5.

**Table A1:** Magnitudes of the differences in angles (|*ψ_D_ − ψ*|) between the diffraction peak (*ψ_D_*) in cases of (0001) or $\left(10\bar{1}1\right)$ orientation and trajectory of out-of-plane grazing-incidence XRD (*ψ*).

	Magnitude of Differences in Angle, (|*ψ_D_ − ψ*|) (deg)	
Diffraction Peak	(0001) Orientation	$\left(10\bar{1}1\right)$ Orientation
0002	17.21	44.29
0004	36.28	25.22
$10\bar{1}0$	74.12	12.52
$10\bar{1}1$	43.49	18.13
$10\bar{1}2$	19.00	4.97
		25.83
$10\bar{1}3$	0.24	1.42
		19.08
$10\bar{1}4$	15.87	3.89
		11.31
$10\bar{1}5$	31.76	1.14
		10.76
$11\bar{2}0$	61.71	12.11
$11\bar{2}2$	24.06	7.87
$11\bar{2}4$	10.61	17.21
$20\bar{2}0$	56.82	4.79
$20\bar{2}1$	40.34	8.96
		21.24
		22.16
$20\bar{2}2$	23.13	13.72
		18.32
		38.48
$20\bar{2}3$	6.16	5.30
		22.60
		34.21
